# Mitigate Cascading Failures on Networks using a Memetic Algorithm

**DOI:** 10.1038/srep38713

**Published:** 2016-12-09

**Authors:** Xianglong Tang, Jing Liu, Xingxing Hao

**Affiliations:** 1 Key Laboratory of Intelligent Perception and Image Understanding of Ministry of Education, Xidian University, Xi’an 710071, China

## Abstract

Research concerning cascading failures in complex networks has become a hot topic. However, most of the existing studies have focused on modelling the cascading phenomenon on networks and analysing network robustness from a theoretical point of view, which considers only the damage incurred by the failure of one or several nodes. However, such a theoretical approach may not be useful in practical situation. Thus, we first design a much more practical measure to evaluate the robustness of networks against cascading failures, termed *R*_cf_. Then, adopting *R*_cf_ as the objective function, we propose a new memetic algorithm (MA) named MA-R_cf_ to enhance network the robustness against cascading failures. Moreover, we design a new local search operator that considers the characteristics of cascading failures and operates by connecting nodes with a high probability of having similar loads. In experiments, both synthetic scale-free networks and real-world networks are used to test the efficiency and effectiveness of the MA-R_cf_. We systematically investigate the effects of parameters on the performance of the MA-R_cf_ and validate the performance of the newly designed local search operator. The results show that the local search operator is effective, that MA-R_cf_ can enhance network robustness against cascading failures efficiently, and that it outperforms existing algorithms.

Many man-made infrastructures such as the Internet, transportation networks, and electric power grids can be represented as complex networks^1^. Because these complex networks play an important role in society, their robustness is pivotal^2^,^3^,^4^. However, most of these infrastructures have been found to be heterogeneous and to have a power-law degree distribution^1^,^5^,^6^. With their “heavy-tailed” properties, these complex networks have been found to be robust against random attacks; however, they are rather fragile under malicious attacks, especially cascade-based attacks^7^,^8^.

Cascading failures are common in modern social networks. For example, in electrical power grids, when a power transmission station or a power line goes down, its power will be shifted to the nearby stations (lines). In most cases, neighbouring stations can manage the extra load. However, in some extreme circumstances, these neighbouring stations may become overloaded and fail, resulting in a redistribution of their loads to their neighbours. Ultimately, the redistribution effort may lead to a cascading failure in which a large number of power transmission stations (lines) are overloaded and, consequently, malfunction^9^. Cascading failures may also take place on the Internet. The load on an Internet router represents data packets that must be transmitted per unit of time, and overloading corresponds to congestion^10^. Rerouting data packets from a congested router to another might spread the congestion to a large fraction of subnetworks. Some Internet collapses have been caused by congestion^11^. Another example is a power grid, in which each component is designed to deal with a specific load of power. On August 14, 2003 in Canada and the northeastern United States, a massive power blackout occurred that led to a cascading failure^12^. A similar breakdown occurred in southern Oregon on August 10, 1996 13,14.

Cascading failures in complex networks have been widely studied over the past few decades^15^,^16^,^17^,^18^,^19^,^20^,^21^,^22^,^23^,^24^. Different cascading failure models have been proposed to reproduce cascading phenomena. Motter *et al*. first proposed the “*C-L*” model in ref. 18, performing experiments on both random and scale-free networks that focused on cascading triggered by the failure of a single node. The “*C-L*” model obtained good results that were consistent with experts’ intuition about how cascading failures occur. Crucitti *et al*.^19^ introduced a dynamical model that considered the dynamical redistribution of flow in networks, in which overloaded nodes obstruct network traffic rather than removed. Zhao *et al*.^20^ provided a mathematical proof of the “*C*-*L*” model in scale-free networks that analysed the cascading breakdown in scale-free networks in terms of phase transitions. Feng *et al*.^23^ proposed an approach of simple, self-consistent probability equations to study cascading behaviours in interdependent networks and showed that this approach can greatly simplify the mathematical analysis of systems ranging from single-layer networks to various types of interdependent networks. Hu *et al*.^24^ used a percolation approach to study more realistic coupled networks system in which both interdependent and interconnected links exist and found rich and unusual phase-transition phenomena—including mixed first- and second-order hybrid transitions.

Based on different cascading failure models, various strategies have been proposed to enhance network robustness against cascading failures. Koç *et al*.^25^ proposed a robust metric for cascading failures on power grid networks; an entropy-based metric was introduced in ref. 26. Wang *et al*.^27^ studied cascading failures on the Internet. Based on a new cascading edge model, they proposed some methods to protect the Internet from cascading failures. However, all the above methods focused only on cascades triggered by removing one or two nodes, and such methods cannot evaluate the overall robustness of networks against cascading failures and may not be useful in many practical applications. Additionally, these methods rarely take the cost involved in updating the real-world systems into account.

Considering only the cascading failure resulting from removing individual nodes in networks is insufficient because many of the remaining nodes are still connected; therefore, the network still maintains its integrity to a certain extent. In contrast, in this paper, we first design a new robustness measure to evaluate the overall robustness of networks against cascading failures. In this robustness measuring scheme, the network is attacked through cascading failures repeatedly until the entire network collapses. During this process, after each cascade attack, the remaining large network components are calculated.

Based on this measure, we propose a memetic algorithm (MA) called MA-R_cf_ that enhances network robustness against cascading failures. Memetic algorithms form a popular branch of evolutionary algorithms (EAs) that successfully combine global and local searches and have been shown to be more efficient and more effective than traditional EAs for many problems^28^,^29^,^30^. In a previous study, we designed a new memetic algorithm named MA-RSF_MA_, which improves the robustness of scale-free networks against malicious attacks that achieved a good performance^31^. Thus, the algorithm proposed here, MA-R_cf_, is based on the framework developed for MA-RSF_MA_ and makes use of the properties of cascading failures to design new operators; that is, a new local search operator that considers the characteristic of cascading failures is designed for MA-R_cf_. In MA-R_cf_, the degree distribution of networks is also kept unchanged to minimize the costs of updating real-world systems. Both synthetic and real-world networks are used to validate the performance of the MA-R_cf_. The experimental results show that the MA-R_cf_ can enhance the network robustness against cascading failures efficiently. Moreover, some properties of robust networks are also analysed.

## Methods

### Robustness Measure for Cascading Failures

A network can be modelled as a graph, *G* = (*V, E*), where *V* = {1, 2, …, *N*} is a set of *N* nodes and *E* = {*e*_*jk*_| *j, k*∈*V* and *j* ≠ *k*} is a set of *M* links. In ref. 18, Motter *et al*. proposed the “*C-L*” model for cascading failures in which, for a given network, at each time step, one unit of the relevant quantity (such as energy or goods) is exchanged between each pair of nodes and transmitted along the shortest connecting path. The “load” at a node consists of the total number of shortest paths passing through it^32^,^33^. Each node carries the maximum load that it can handle, and in man-made networks, node capacity is limited by economic costs. The capacity *C*_*i*_ of node *i* and its initial load *L*_*i*_ have the following proportional relation:


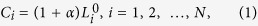


where the constant *α* is a tolerance parameter, and 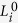 is the initial load of the *i*th node. Initially, the network operates in a free-flow state insofar as *α* ≥ 0. However, the failure of a node for any reason triggers the dynamics of the redistribution of loads. When the load at a node becomes larger than the node’s capacity, the node fails. This forces the load previously carried by that node to shift to its neighboring nodes, which in turn, can cause them to fail. Consequently, subsequent failures can occur, and this step-by-step process is a cascading failure^18^.

In ref. 34, Schneider *et al*. proposed an effective robustness measure, *R*, to evaluate networks’ ability to resist targeted attacks on individual nodes. The *R* measure is based on the “giant component,” namely, the largest connected component left in the network after each node removal. To calculate *R*, the network must be attacked until only separated nodes are left. Thus, *R* can evaluate the robustness of entire networks. Therefore, we combine the “*C-L*” model with *R* to design a new measure, *R*_*cf*_, which can evaluate the overall robustness of networks against cascading failures. With the original property of “*C-L*” model in mind, the process for calculating *R*_*cf*_ is described below.

Step 1. ***S***_***sum***_ ← 0 and ***t*** ← 1, where ***S***_***sum***_ is the accumulated size of the giant components and ***t*** is the index of cascaded attack rounds;

Step 2. Calculate the initial load 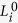 and ***C***_***i***_ of each node;

Step 3. Remove the node with the maximum load and all the edges connected to it;

Step 4. If the number of remaining nodes is equal to 1, go to Step 6;

Step 5. Recalculate the 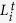 of each remaining node:

If a remaining node ***i*** is overloaded, namely, ***L***_***i***_ > ***C***_***i***_, then remove node ***i*** and the edges connected to it, then, go to Step 4;

If no remaining node is overloaded, calculate the relative size of the giant component ***S***^***t***^, ***S***_***sum***_ **←** ***S***_***sum***_**+*****S***^***t***^, ***t*** **←** ***t*** **+** **1**, then, go to Step 3;

Step 6. Calculate the robustness measure ***R***_***cf***_ against cascading failures as follows,


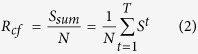


where *T* is the total number of rounds that a cascading failure-based attack needs to destroy the entire network, reducing it to only one node. Obviously, *T* may vary even for networks of the same size; therefore, the normalization factor 1/*N* ensures comparability for the robustness of networks with different sizes.

### Memetic Algorithm to Enhance R_cf_

Memetic algorithms have been shown to be highly capable of searching for the optimal solution in optimization problems^28^,^29^,^30^. In ref. 31, we designed a new memetic algorithm named MA-RSF_MA_ to improve the robustness of scale-free networks against malicious attacks, and it achieved a good performance^31^. Thus, based on the framework of MA-RSF_MA_, in this paper, we propose a new memetic algorithm, MA-R_cf_, to enhance the overall robustness of networks against cascading failures. By considering the intrinsic property of cascading failures, we design a new local search operator for MA-R_cf_ that takes *R*_*cf*_ as its objective function while keeping the degree of each node unchanged. Next, we introduce the representation of chromosomes and the initialization process. Then, we describe the evolutionary operators, including the newly designed local search operator. Finally, we summarize the entire framework of the MA-R_cf_ algorithm.

#### Representation and initialization

In the MA-R_cf_ algorithm, each chromosome represents a network. Initially, MA-R_cf_ has a population with *W* chromosomes. The initialization process for MA-R_cf_ is the same as that used for MA-RSF_MA_^31^. During the initialization, because we need to preserve the number of links and the degree of each node, each chromosome is generated by randomly adjusting a fraction of the edges in the initial network, *G*_0_—that is, the connections of two randomly chosen edges that have no common nodes are swapped in the network. During the initialization, the goal is to generate different networks with the same degree distribution; therefore, any edge-swapping operations that can keep the network connected are accepted without checking whether the swap improves the robustness of the network. The details of this initialization process are summarized in Algorithm 1 (also refer to ref. 31 for more information).


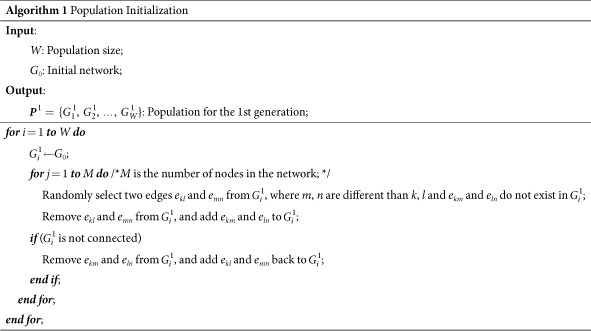


### Evolutionary operators

In evolutionary algorithms, crossover operators are often performed to exchange genetic information among the individuals in the population. In ref. 31, a new crossover operator that operates on complex networks is designed that exchanges the structures of two networks effectively without changing their degree distributions. We also employ this crossover operator in this paper. This crossover operator (whose details are summarized in Algorithm 2) acts on two randomly selected parent chromosomes and generates a pair of child chromosomes. Please refer to ref. 31 for more information about this crossover operator.


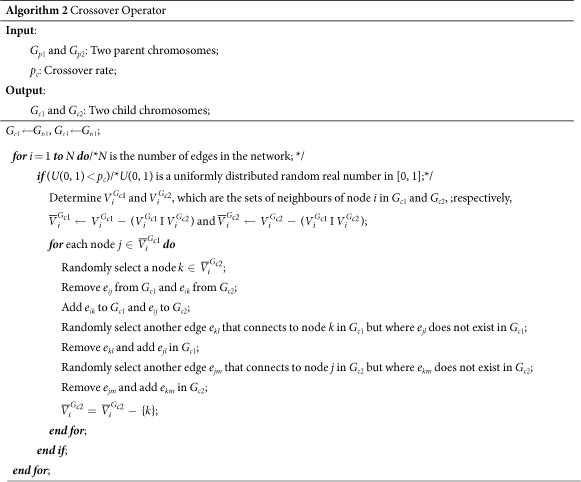


The local search operator is another important operator in MAs. In ref. 35, Tanizawa *et al*. found that networks with an “onion-like” structure, where nodes with almost the same degree are connected to each other, are more robust under targeted attacks than those without such onion-like structures. Inspired by this, to search for networks that are the most robust against cascading failures, we design a new local search operator that lets nodes with similar loads connect to each other. The principle of this operator is simple: if a node with small load connects to a node with a very large load, the small-load node will crash immediately if the large-load node fails, because the small-load node has insufficient capacity to handle the extra load. Therefore, connecting nodes with similar loads to each other have a high probability of avoiding such situations. Suppose edges *e*_*ij*_ and *e*_*pq*_ are selected and are swapped to *e*_*ip*_ and *e*_*jq*_. This swap is accepted only if


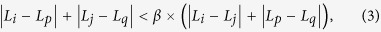


where *L*_*i*_, *L*_*j*_, *L*_*p*_ and *L*_*q*_ are the loads of the corresponding nodes, and *β* is a parameter in the range of [0, 1] that controls the acceptance level for the difference in loads between nodes. The smaller the value of *β* is, the stronger the constraint is and, thus, the larger the reduction in load differences is. Consequently, this operator can effectively guarantee that nodes with similar loads will be connected to each other, which enhances the search for load-balanced networks. The details of this local search operator are given in Algorithm 3.


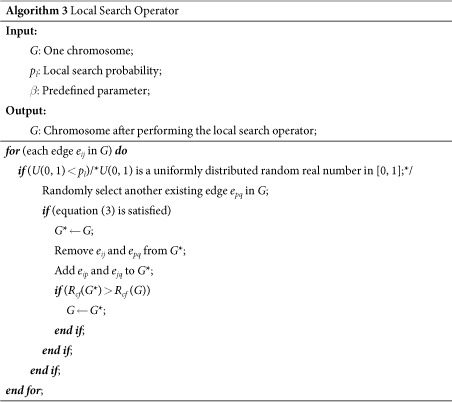


MA-R_cf_ uses binary tournament selection in each generation to select the chromosomes for the next population. Binary tournament selection involves a “tournament” between two chromosomes chosen randomly from the population in which the winner is the chromosome whose fitness is better. Binary tournament selection is a popular method for selecting better chromosomes from a population in an evolutionary algorithm.


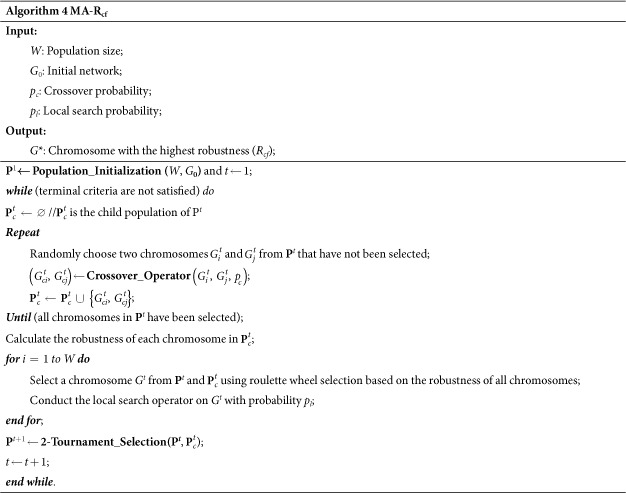


#### Implementation of MA-R_cf_

In MA-R_cf_, the initialization operator is first used to generate an initial population with *W* chromosomes. In each generation, the crossover operator is applied to the population first, and then, the local search operation is conducted. After performing the crossover operator, a new child population is obtained. Then, the local search operator and the binary tournament selection operator are applied to both the parent and child populations to generate the child population for the next generation. Finally, the best chromosome in the last population is the most robust network found. The framework of MA-R_cf_ is summarized in Algorithm 4.

## Results

In this section, because scale-free networks have become an important type of network, experiments are conducted on both synthetic scale-free networks and real-world networks to validate the performance of MA-R_cf_. We also study some of the network properties of the robust networks obtained by MA-R_cf_. The synthetic scale-free networks were generated using the BA model^5^, and their average degree was set to 4. In ref. 31, Zhou *et al*. showed that MA-RSF_MA_ can improve the robustness of scale-free networks against malicious attacks effectively; consequently, we also compare networks optimized by MA-R_cf_ with those optimized by MA-RSF_MA_ to investigate the different properties of both optimized networks.

The parameter *α* in (1) reflects the capacity of a node to handle its load. A larger *α* indicates a stronger node. The value of *α* is always assumed to be in the range [0, 1]: a *α* > 1 is unrealistically large^18^,^19^. In this work, we assume that ability of a node to handle its loads is average (neither very strong nor very weak). Thus, in the following experiments, *α* is set to a median value, 0.5.

**Figure 1:**
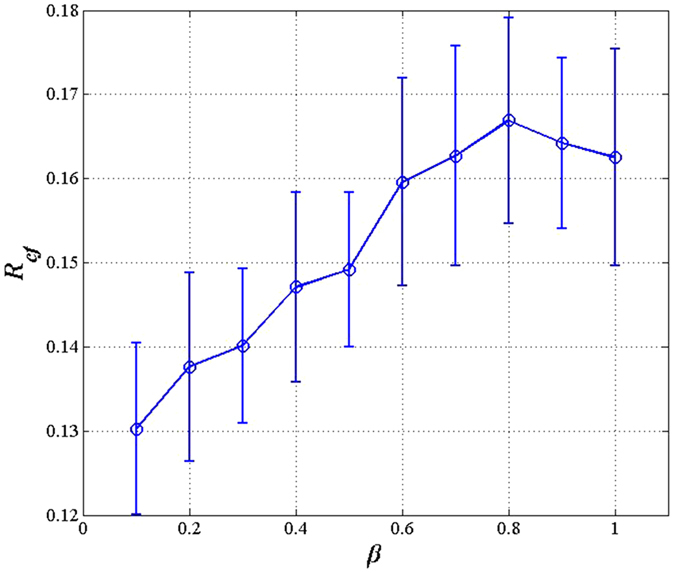
The effect of parameter *β* on the performance of MA-R_cf_.

In the local search operator, the tolerance parameter *β* controls the percentage by which loads can differ between connected nodes. Therefore, we first conducted an experiment to find an appropriate value for *β*. This experiment used BA networks with 100 nodes. The robustness obtained by MA-R_cf_ under different values of *β* is plotted in Fig. 1. The results are averaged over ten independent runs on each sampled point. As Fig. 1 shows, MA-R_cf_ achieves the highest robustness when *β* equals 0.8. Thus, *β* is set to 0.8 in the following experiments.

The other parameters of MA-R_cf_ were set as follows: the population size *W* was set to 10, the crossover probability *p*_*c*_ and the local search probability *p*_*l*_ were set to 0.8 and 0.5, respectively, and the maximum number of objective function evaluations was set to 5 × 10^4^.

### Experiments on Synthetic Networks

In this experiment, scale-free networks with different scales were used to test the performance of MA-R_cf_. First, an experiment was carried out to test the effectiveness of the local search operator. In this experiment, versions of MA-R_cf_ both with and without the local search operator were tested on BA networks with 100, 200, 300, and 500 nodes. The obtained robustness values are listed in Table 1, which shows that the MA-R_cf_ version with the local search operator always performs better than the version without the local search operator. Therefore, the local search operator in MA-R_cf_ is effective.

Next, some experiments were conducted to test the ability of MA-R_cf_ to search for the most robust networks. Network structure optimization is a hard optimization problem. In existing works, the hill climbing algorithm^34^ and the simulated annealing algorithm^36^,^37^ are widely used to address this problem. Thus, we compared the performance of MA-R_cf_ with that of the hill climbing algorithm^34^ and the simulated annealing algorithm^36^,^37^. The maximum number of objective function evaluations for these two algorithms was also set to 5 × 10^4^ to obtain the results of these three algorithms at the same computational cost.

**Figure 2:**
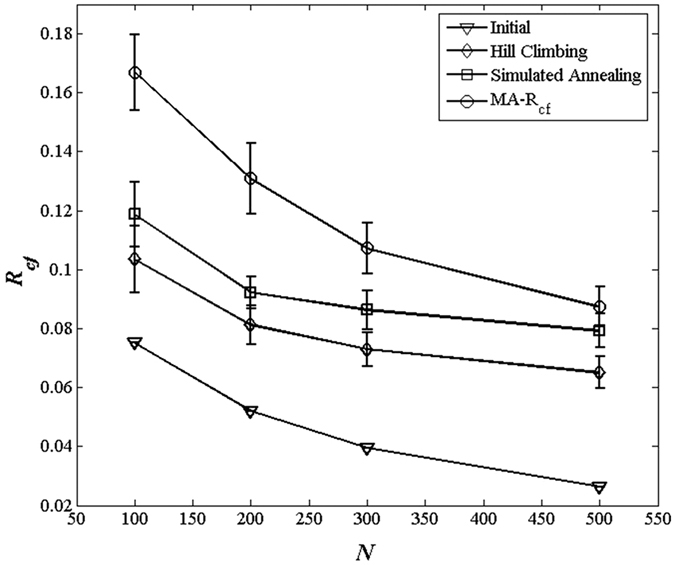
A comparison between MA-R_cf_ and existing algorithms on BA networks of different sizes.

We tested BA networks with 100, 200, 300, and 500 nodes. The best, worst and average values of *R*_*cf*_ of the three algorithms over 10 independent runs are reported in Table 2. In addition, the corresponding curves of the average robustness obtained by the different algorithms are plotted in Fig. 2. As shown, MA-R_cf_ obtains the highest robustness values among these algorithms on all test networks. That is, MA-R_cf_ always finds more network structures more robust to cascading failures than do the other algorithms.

**Figure 3:**
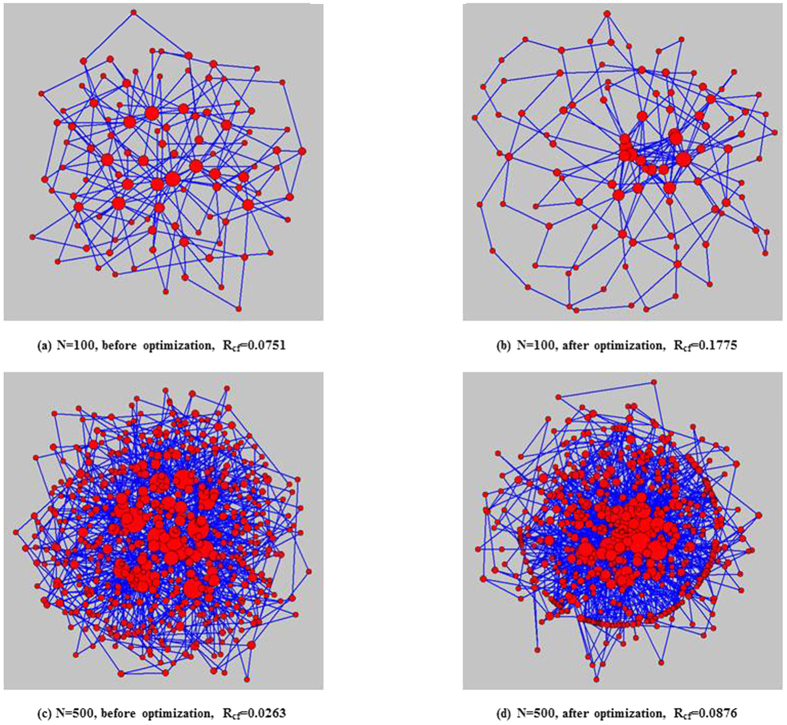
The network topology before and after optimization by MA-R_cf_. The size of each node is proportional to its degree.

It is useful to study the robustness of the network structures obtained MA-R_cf_. Thus, the network topologies of BA networks before and after optimized by MA-R_cf_ are plotted in Fig. 3, where the size of each node is proportional to its degree. As shown, before optimization, low degree nodes are often connected to nodes with high degrees; consequently, the entire network is composed of numerous star networks with hub nodes. However, the optimized networks which have higher *R*_*cf*_, the low degree nodes are more likely to be connected to other low degree nodes and the high degree nodes are more likely to be connected to other high degree nodes. The entire network is a hub-node-connected structure. Considering the property of cascading failures, it is easy to understand why hub-node-connected networks have a stronger ability to resist cascade failures: when a hub node fails, the neighbouring hub nodes can withstand the additional loads effectively, preventing the spread of cascading failures.

**Figure 4:**
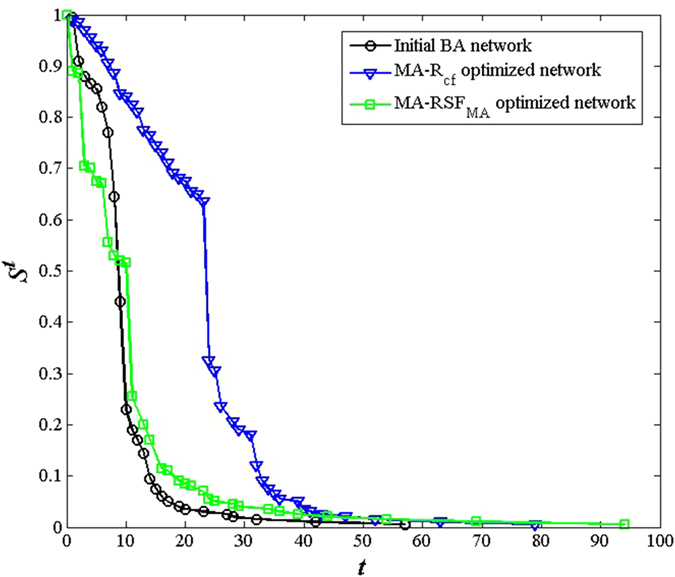
The change in the relative size of the giant component *S*^*t*^ over a series of **cascaded attack circles** ***t***. The BA network has 200 nodes. The *R*_*cf*_ values of the initial BA network, the optimized network obtained by MA-RSF_MA_ and the optimized network obtained by MA-R_cf_ were 0.0521, 0.0558 and 0.1319, respectively.

Next, we carried out an experiment to test how well the networks obtained by MA-R_cf_ resist cascading failures. We simulated the process of cascaded failures on BA networks with 200 nodes until the size of the giant component decreased to 1. The decreasing progress of the size of the giant component *S*^*t*^ is plotted in Fig. 4. As shown, along with the increasing cascade attack circle *t*, the MA-R_cf_ optimized network, which has a higher *R*_*cf*_ value, protects the giant component more effectively. The area between the curve of the “Initial BA network” and the curve of the “MA-R_cf_ optimized network” in Fig. 4 represents the amount of cascade failure mitigation, which corresponds to improving network robustness against cascade failures by 153%. These results show that the networks obtained by MA-R_cf_ can resist cascading failures effectively.

**Figure 5:**
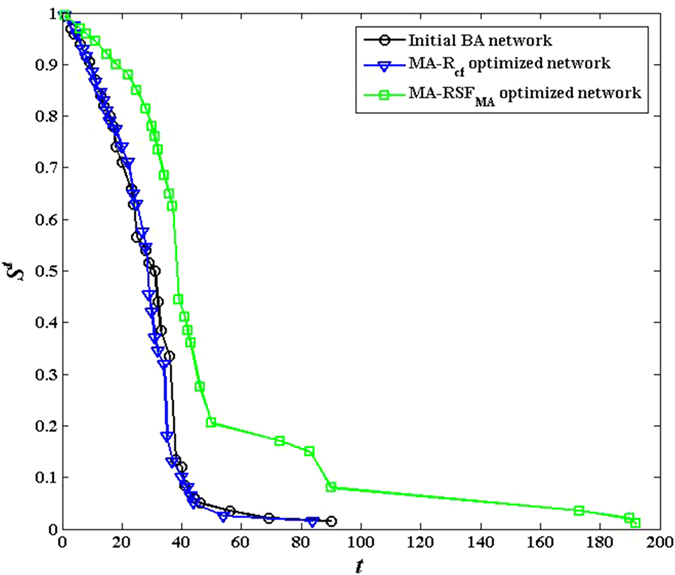
The change in the relative size of the giant component *S*^*t*^ under **high node degree attack circles** ***t***. The BA network has 200 nodes. The *R*_*cf*_ values of the initial BA network, the optimized network obtained by MA-RSF_MA_ and the optimized network obtained by MA-R_cf_ were 0.0521, 0.0558 and 0.1319, respectively.

In ref. 31, Zhou *et al*. found that the onion-like network in which nodes with similar degree connect to each other can contribute to resisting high node degree attacks, we plotted the decreasing process of the size of *S*^*t*^ of networks optimized by MA-RSF_MA_ under cascading failures in Fig. 4, which has 200 nodes. In Fig. 5, we separately plotted the decreasing process of *S*^*t*^ of networks optimized by both MA-R_cf_ and MA-RSF_MA_ under high node degree attacks. In each attack circle, the node with largest degree and all the edges connected to it are removed. To perform a fair comparison, the parameters for MA-RSF_MA_ were set to the same as those for MA-R_cf_, namely, the population size was set to 10, the crossover probability and the local search probability were set to 0.8 and 0.5, respectively, and the maximum number of objective function evaluations was set to 5 × 10^4^. In Fig. 4, under cascaded attack circles, the size of the *S*^*t*^ of the MA-RSF_MA_ optimized networks decreases as fast as that of the initial BA network—even more sharply after the first several attack rounds. Moreover, under high node degree attack circles, the size of the *S*^*t*^ of the MA-R_cf_ optimized network decreases as fast as initial BA networks (see Fig. 5 for more details). In other words, the MA-R_cf_ algorithm cannot contribute to resisting high node degree attacks. These two experiments show that although the network structures optimized by these two algorithms have some similarity, their ability to resist cascading failures is significantly different.

We are also interested in whether other important network properties might have changed because of the optimization. Therefore, in Table 3 shows the results of assessing the assortativity coefficient^38^, the average shortest path length and the global communication efficiency^39^ of networks both before and after being optimized by MA-R_cf_. As shown, before the optimization, the BA networks have negative assortativity coefficient values that become positive after the optimization. In other words, the correlation degree of the networks changes from disassortativeness to assortativeness after the optimization. This occurs because the optimization process promotes the connection of high degree nodes with other high degree nodes. After the optimization, the average shortest path length of BA networks is slightly increased while the global communication efficiency has a slight decrease, which means that the optimization process has no significant effect on network communication efficiency.

Because the networks obtained by MA-RSF_MA_ are also assortative, it is interesting to study the difference in terms of the network properties of networks obtained by both MA-R_cf_ and MA-RSF_MA_; these properties are listed in Table 3. We can see that when both algorithms optimize the same network, the network obtained by MA-R_cf_ is far less assortative than that obtained by MA-RSF_MA_. In addition, the average shortest path length of the MA-R_cf_ is only half that of MA-RSF_MA_. Moreover, the networks obtained by MA-R_cf_ have higher global communication efficiency.

### Experiments on Real World Networks

In this section, MA-R_cf_ is applied to two real-world networks. One is an electrical power grid in Western Europe (mainly Portugal and Spain)^40^, labelled the WE Power grid network. It has 217 nodes and 320 edges. The average degree of the WE Power grid network is 2.95. The other network is the US air network—the US air transportation system^41^—consisting of 332 airports and 2126 air routes, in which the nodes represent airports and the edges present flights. The average degree of the US air network is 12.81. These two real networks are well connected and without any separate component.

**Figure 6:**
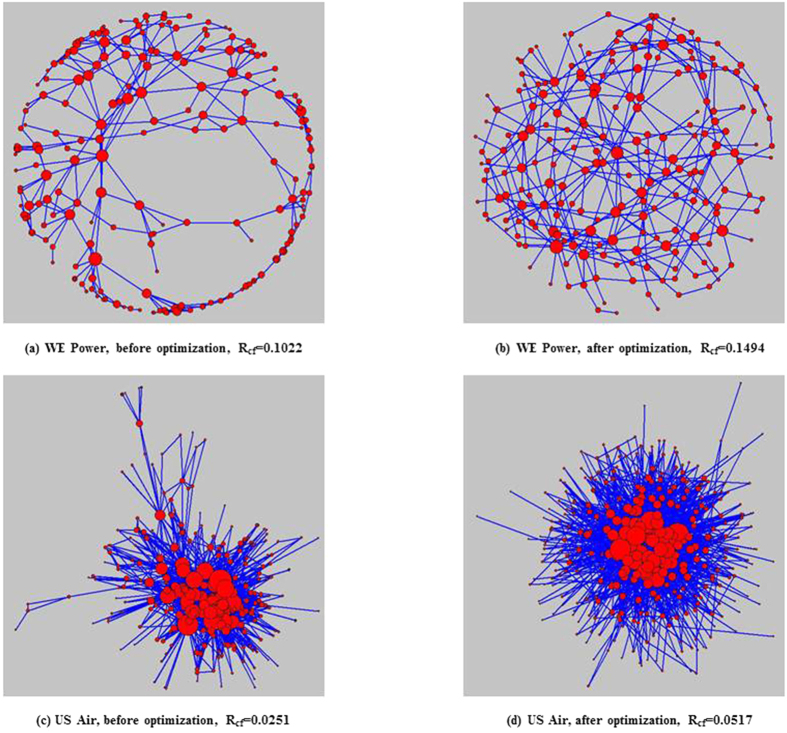
The network topology of two real world networks before and after optimization by MA-R_cf_. The size of each node is proportional to its degree.

The hill climbing algorithm, simulated annealing algorithm and MA-R_cf_ are used to independently optimize the above two networks. The obtained robustness values are reported in Table 4. As we can see, MA-R_cf_ always performs better than the two other algorithms. The network topologies of these two real networks before and after optimized by MA-R_cf_ are shown in Fig. 6. Comparing the network structure before and after optimization, we can see that, in the optimized networks, nodes with similar degrees are more likely to connect with each other, and the hub nodes are more closely connected to each other than before. Consequently, even for an existing network, MA-R_cf_ can find a structure more robust against cascading failure. By comparing the robust structure with the initial structure, we can find several key edges, which—if changed—would increase network robustness significantly. Considering the cost of optimization, there is no need to change all the edges of the real network; instead, MA-R_cf_ can help find the key edges.

The assortativity coefficient, average shortest path length and global communication efficiency of these two real networks both before and after optimization are reported in Table 5. As listed, the WE Power network has a positive assortativity coefficient while the US air network has a negative assortativity coefficient. After optimization by MA-R_cf_, the WE Power network has stronger assortativeness, while the disassortativeness of the US air network gets weaker. This result is the same as the results of the experiments with synthetic networks, further verifying that networks with more hub nodes connected to each other have a stronger ability to resist cascading failures. Interestingly, after optimization by MA-R_cf_, the average shortest path length of these two networks decreases while their global communication efficiency increases. In other words, MA-R_cf_ can not only increase a network’s global robustness against cascading failures but can also increase its global communication efficiency—even when applied to real networks.

## Discussions

Securing network infrastructure is critical in today’s society. When studying networks subject to cascading failures, considering only the damage from one or even several nodes is insufficient. In this paper, we describe the design of a more comprehensive index that can evaluate the ability of networks to resist cascading failures. To enhance networks resistance to cascading failures, we propose a memetic algorithm, MA-R_cf_. Then, to test the performance of MA-R_cf_, we tested it on both synthetic and real networks. The topologies of the robust networks obtained by MA-R_cf_ are shown and some of their network properties are discussed. From experiments comparing with MA-R_cf_ other network optimization algorithms, we can conclude that MA-R_cf_ achieves a better performance, showing that MA-R_cf_ is an effective algorithm for enhancing the robustness of networks against cascading failures.

When dealing with complex networks, the large computational complexity of calculating shortest paths limits the algorithms that rely on such calculations from being applied to large-scale networks. For example, the computation of R_cf_ in Equation (2) needs to calculate the shortest path of the network under each cascading attack circle; consequently, MA-R_cf_ is unable to optimize large networks at a low computational cost. However, studying the effects of MA-R_cf_ on cascading failures is still meaningful. MA-R_cf_ provides an opportunity to explore network structures that are robust against cascading failures. In this paper, we apply MA-R_cf_ to networks with specific sizes and study the topological structure and network properties of robust networks. The experiments show connecting hub nodes to each other more closely would be a good strategy when designing networks that are robust against cascading failures. Moreover, extending this discovery to large-scale networks is not difficult. In contrast with other traditional algorithms, MA-R_cf_ is a competitive algorithm for optimizing networks against cascading failures.

## Additional Information

**How to cite this article**: Tang, X. *et al*. Mitigate Cascading Failures on Networks using a Memetic Algorithm. *Sci. Rep.* **6**, 38713; doi: 10.1038/srep38713 (2016).

**Publisher's note:** Springer Nature remains neutral with regard to jurisdictional claims in published maps and institutional affiliations.

## Figures and Tables

**Table 1:** The *R*_*cf*_ values of BA networks obtained by MA-R_cf_ with and without the local search operator.

N	Initial	Without (Average ± Standard Deviation)	With (Average ± Standard Deviation)
100	0.0751	0.1617 ± 0.0135	0.1669 ± 0.0128
200	0.0521	0.1202 ± 0.0127	0.1309 ± 0.0119
300	0.0395	0.0924 ± 0.0097	0.1072 ± 0.0085
500	0.0263	0.0783 ± 0.0074	0.0874 ± 0.0067

The results are averaged over 10 independent runs.

**Table 2:** The *R*_*cf*_ of BA networks of different sizes obtained by the three tested algorithms.

N	Algorithms	Best	Worst	Average ± Standard deviation
100	Hill Climbing	0.1231	0.0912	0.1035 ± 0.0113
	Simulated Annealing	0.1356	0.1037	0.1187 ± 0.0109
	**MA-R**_**cf**_	0.1797	0.1476	**0.1669** ± **0.0128**
200	Hill Climbing	0.0926	0.0747	0.0813 ± 0.0066
	Simulated Annealing	0.1021	0.0893	0.0922 ± 0.0053
	**MA-R**_**cf**_	0.1427	0.0891	**0.1309** ± **0.0119**
300	Hill Climbing	0.0821	0.0668	0.0729 ± 0.0059
	Simulated Annealing	0.0932	0.0720	0.0864 ± 0.0066
	**MA-R**_**cf**_	0.1157	0.0892	**0.1072** ± **0.0085**
500	Hill Climbing	0.0782	0.0595	0.0651 ± 0.0054
	Simulated Annealing	0.0883	0.0692	0.0793 ± 0.0059
	**MA-R**_**cf**_	0.1021	0.0722	**0.0874** ± **0.0067**

The results are averaged over 10 independent runs.

**Table 3:** The changes in important network properties of BA networks with different sizes before and after optimization by MA-R_cf_ and MA-RSF_MA_, including the assortative coefficients (A), the average shortest path length (L) and the global communication efficiency (C).

N		A (Average ± Standard Deviation)	L (Average ± Standard Deviation)	C (Average ± Standard Deviation)
100	Before Optimization	−0.1489 ± 0.0000	3.1681 ± 0.0000	0.3563 ± 0.0000
	MA-RSF_MA_	0.6206 ± 0.0242	8.1168 ± 0.2203	0.2186 ± 0.0093
	MA-R_cf_	0.3956 ± 0.0132	3.8307 ± 0.1841	0.3139 ± 0.0125
200	Before Optimization	−0.2194 ± 0.0000	3.5645 ± 0.0000	0.3124 ± 0.0000
	MA-RSF_MA_	0.3951 ± 0.0176	7.9266 ± 0.2312	0.2057 ± 0.096
	MA-R_cf_	0.1734 ± 0.0118	3.5726 ± 0.1873	0.3115 ± 0.0103
300	Before Optimization	−0.2344 ± 0.0000	3.6327 ± 0.0000	0.3001 ± 0.0000
	MA-RSF_MA_	0.3480 ± 0.0198	8.2787 ± 0.2241	0.1801 ± 0.0102
	MA-R_cf_	0.1470 ± 0.0093	3.7308 ± 0.1254	0.2943 ± 0.0089
500	Before Optimization	−0.2507 ± 0.0000	3.8264 ± 0.0000	0.2836 ± 0.0000
	MA-RSF_MA_	0.3651 ± 0.0142	8.1968 ± 0.2101	0.1869 ± 0.0114
	MA-R_cf_	0.1033 ± 0.0076	3.8304 ± 0.1219	0.2814 ± 0.0081

The values of the optimized networks were averaged over 10 independent runs.

**Table 4:** The robustness of networks after optimization with different algorithms on two real world networks.

Network	Algorithms	Best	Worst	Average ± Standard deviation
WE Power	Hill Climbing	0.1221	0.1072	0.1135 ± 0.0063
	Simulated Annealing	0.1256	0.1097	0.1187 ± 0.0059
	**MA-R**_**cf**_	0.1494	0.1126	**0.1330** ± **0.0082**
US Air	Hill Climbing	0.0463	0.0378	0.0415 ± 0.0047
	Simulated Annealing	0.0481	0.0382	0.0422 ± 0.0051
	**MA-R**_**cf**_	0.0537	0.0401	**0.0475** ± **0.0062**

The results shown were averaged over 10 independent runs. The initial *R*_*cf*_ values of the WE Power network and US air network were 0.1022 and 0.0251, respectively.

**Table 5:** The changes in some important network properties of real world networks before and after optimization by MA-R_cf_, including the assortative coefficients (A), the average shortest path length (L) and the global communication efficiency (C).

N		A (Average ± Standard Deviation)	L (Average ± Standard Deviation)	C (Average ± Standard Deviation)
WE Power	Before Optimization	0.1269 ± 0.0000	6.9381 ± 0.0000	0.1870 ± 0.0000
	After Optimization	0.2176 ± 0.0097	4.9511 ± 0.1903	0.2313 ± 0.0115
US Air	Before Optimization	−0.2078 ± 0.0000	2.7381 ± 0.0000	0.4059 ± 0.0000
	After Optimization	−0.0819 ± 0.0056	2.4980 ± 0.1410	0.4336 ± 0.0089

The values of optimized networks were averaged over 10 independent runs.
